# Fate of systemically and locally administered adipose‐derived mesenchymal stromal cells and their effect on wound healing

**DOI:** 10.1002/sctm.19-0091

**Published:** 2019-10-15

**Authors:** Karlien Kallmeyer, Dominik André‐Lévigne, Mathurin Baquié, Karl‐Heinz Krause, Michael S. Pepper, Brigitte Pittet‐Cuénod, Ali Modarressi

**Affiliations:** ^1^ Department of Plastic Reconstructive & Aesthetic Surgery, University Hospitals of Geneva, University of Geneva Geneva Switzerland; ^2^ Institute for Cellular and Molecular Medicine (ICMM), Department of Immunology, and SAMRC Extramural Unit for Stem Cell Research and Therapy University of Pretoria Pretoria South Africa; ^3^ Department of Pathology and Immunology University of Geneva Geneva Switzerland; ^4^ Department of Human Genetics and Development University of Geneva Geneva Switzerland; ^5^ Neurix SA Geneva Switzerland

**Keywords:** adipose‐derived mesenchymal stromal cells, bioluminescence imaging, firefly luciferase, green fluorescent protein, in vivo imaging, wound healing, wound repair

## Abstract

There is increasing interest in the use of adipose‐derived mesenchymal stromal cells (ASCs) for wound repair. As the fate of administered cells is still poorly defined, we aimed to establish the location, survival, and effect of ASCs when administered either systemically or locally during wound repair under physiological conditions. To determine the behavior of ASCs, a rat model with wounds on the dorsal aspect of the hind paws was used and two treatment modes were assessed: ASCs administered systemically into the tail vein or locally around the wound. ASCs were transduced to express both firefly luciferase (Fluc) and green fluorescent protein to enable tracking by bioluminescence imaging and immunohistological analysis. Systemically administered ASCs were detected in the lungs 3 hours after injection with a decrease in luminescent signal at 48 hours and signal disappearance from 72 hours. No ASCs were detected in the wound. Locally administered ASCs remained strongly detectable for 7 days at the injection site and became distributed within the wound bed as early as 24 hours post injection with a significant increase observed at 72 hours. Systemically administered ASCs were filtered out in the lungs, whereas ASCs administered locally remained and survived not only at the injection site but were also detected within the wound bed. Both treatments led to enhanced wound closure. It appears that systemically administered ASCs have the potential to enhance wound repair distally from their site of entrapment in the lungs whereas locally administered ASCs enhanced wound repair as they became redistributed within the wound bed.

## Graphical abstract

Adipose‐derived mesenchymal stromal cells were administered either systemically or locally in a rat model of wound repair under physiological conditions. It appears that systemically administered adipose‐derived mesenchymal stromal cells have the potential to enhance wound repair distally from their site of entrapment in the lungs whereas locally administered adipose‐derived mesenchymal stromal cells enhanced wound repair as they became redistributed within the wound bed.

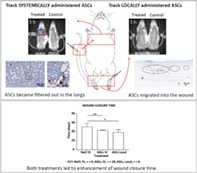

**Significance statement**

The exogenous administration of adipose‐derived mesenchymal stromal cells (ASCs) holds promise as a treatment strategy for wound healing by promoting tissue repair and regeneration. However, the best route of administration is still not well defined. Herein, this article describes the biodistribution and survival of systemically vs locally administered ASCs. Interestingly, both routes of administration led to enhanced wound repair as seen by earlier wound closure. Systemically administered ASCs have the potential to enhance wound repair distally from their site of entrapment in the lungs, whereas locally administered ASCs migrate into the wound bed.

## 1 INTRODUCTION

In the field of regenerative medicine, cell‐based therapies utilizing human mesenchymal stromal cells (MSCs) have generated a great deal of interest for wound healing.^1^, ^2^, ^3^ The exact mechanism underlying the therapeutic benefits of MSCs has yet to be fully elucidated. Their role in tissue maintenance, repair, and regeneration is suggested to stem from their multipotent differentiation capacity, immunomodulatory properties, antimicrobial effect, paracrine signaling, and their ability to migrate to sites of injury.^2^, ^4^, ^5^, ^6^ Although MSCs were originally isolated from bone marrow (BM),^7^ a source used with increasing frequency is subcutaneous adipose tissue.^4^ Both BM‐MSCs and adipose‐derived MSCs (ASCs) are candidate cell types for treating wounds; laboratory and clinical studies, however, favor BM‐MSCs.^3^, ^8^ ASCs are considered to be a potentially useful cell population because of their availability, ease of harvest, high cell yield, and ability to be expanded in culture for clinical use.^4^, ^9^, ^10^, ^11^

MSCs are believed to migrate to damaged tissue in a process called homing.^12^ *In vitro*, MSCs are attracted by cytokines and chemokines that are known to be upregulated under conditions of inflammation.^13^, ^14^, ^15^ However, in vivo, homing of MSCs has not been unequivocally shown. Some studies have reported homing of systemically administered MSCs in mouse models of type 2 diabetes,^16^ traumatic brain injury,^17^ and burn injury,^18^ whereas others reported intrapulmonary cell trapping^19^ and minimal evidence for homing.^20^ Where recirculation of MSCs was shown to occur after becoming trapped in the lungs, only a low percentage of MSCs could be found at the injury site or in non‐target organs.^20^, ^21^, ^22^, ^23^ Most of these studies evaluated the homing of BM‐MSCs. Although BM‐MSCs and ASCs share biological characteristics, differences in their phenotype, differentiation potential, gene expression profile, and immunomodulatory activity have been noted.^11^, ^24^, ^25^ Thus, differences in their homing capacity may also exist, establishing a need to investigate the homing of ASCs and compare this to available BM‐MSCs homing data.

Commonly suggested routes of MSC transplantation include systemic and local injection.^19^, ^26^, ^27^, ^28^, ^29^ Local injection relies on the activity of locally transplanted MSCs at the target site. Systemic injection requires circulating MSC to exit the blood vessels and to migrate to the target site. However, whether administered systemically or locally, where the cells go to and whether they can bind, engraft and even survive in vivo, is still not well understood, particularly for ASCs. Insights into the basic biology of administered ASCs, their biodistribution and their engraftment into damaged tissue, need to be understood to achieve clinical efficiency.

In this study, we evaluate the distribution and survival of ASCs in vivo when administered either systemically or locally using a standardized rat model of cutaneous wound repair under physiological conditions. Defining the location of administered ASCs in vivo is complicated by the lack of a single identifying marker. *In vitro*, ASCs are characterized by their co‐expression of a panel of surface markers; however, this is not possible to achieve in vivo.^30^ Molecular imaging offers the opportunity to track cells in intact organisms. Fluorescent reporters, such as green fluorescent protein (GFP), do not give a sufficiently strong signal to allow for efficient detection in vivo.^31^ A useful method for in vivo cell tracking in small animal models is bioluminescence imaging (BLI).^32^, ^33^, ^34^ BLI allows for non‐invasive imaging over a longer period and provides information on the viability^35^ of cells after transplantation. In this study, we used both BLI and GFP to enable in vivo tracking and post‐mortem identification, respectively.

The therapeutic potential of MSCs for cutaneous wound repair is being investigated in clinical trials for burn wounds,^36^, ^37^ diabetic foot ulcers,^37^, ^38^, ^39^ and critical limb ischemia.^40^, ^41^ Although preclinical and clinical studies using MSCs to enhance wound repair have shown positive outcomes, the benefit of MSC administration for wound healing has not yet been clearly established.^39^, ^42^, ^43^, ^44^, ^45^ As a secondary aim, we evaluated the effect of different modes of ASC administration on wound closure (WC) time. Information is provided on the biodistribution of ASCs and their tissue‐specific location post administration, as well as their effect on WC time. By understanding the fate of administered ASCs in response to injury, this study contributes to establishing the optimal mode of administration required in the clinical setting.

## 2 MATERIALS AND METHODS

All animal experiments were approved by the local veterinary authority (authorization number GE/70/18).

### 2.1 Isolation and culture of primary rat ASCs

Female Wistar rats (≥10 weeks old, ≥250 g, *n* = 14, Janvier labs, Le Genest‐Saint‐Isle, France) were sacrificed by intraperitoneal (IP) injection of 150 mg/kg sodium pentobarbital (Esconarkon AD US. VET., Streuli Pharma, Uznach, Switzerland) followed by excision of the inguinal subcutaneous adipose tissue. The stromal vascular fraction (SVF) was isolated as previously described and plated into a flask (NUNC, Kamstrupvej, Denmark) overnight at 37°C, 5% CO_2_ in high glucose Dulbecco's Modified Eagle's Medium (DMEM 1× + GlutaMAX, 4.5 g/L glucose) supplemented with 20% fetal bovine serum (FBS) and 1% penicillin (10 000 units/mL)‐streptomycin (10 000 μg/mL; pen/strep; Gibco, Life Technologies, NY).^46^, ^47^, ^48^ After 24 hours, non‐adherent cells were removed and the medium changed to complete growth medium (CGM, high glucose DMEM supplemented with 10% FBS and 1% pen/strep). Isolated cells were maintained in CGM (37°C, 5% CO_2_) until 80% confluent before being trypsinized. Cells were counted using the trypan blue dye exclusion assay^49^ and replated as passage 1 (P1) at a density of 5 × 10^3^ cells/cm^2^.

### 2.2 Transduction of ASCs

ASCs were transduced with a dual lentivector expressing GFP and firefly luciferase (Fluc), pCWX‐UBI‐Fluc‐PGK‐GFP. To determine the amount of lentivector needed to transduce greater than 70% of the cells, a multiplicity of infection (MOI) of 0, 2, 5, and 10 was tested (*n* = 4). A MOI of 10 was used for all further experiments. ASCs at P1/P2 were plated at 5 × 10^3^ cells/cm^2^ and allowed to adhere for 24 hours. Lentivectors were added and the cultures left for 72 hours before replacing the medium with fresh CGM. At 80% confluence, ASCs were trypsinized and an aliquot prepared for flow cytometric analysis (*n* = 6) as described below to determine their immunophenotype and the percentage of ASCs expressing GFP. To determine whether ASCs also expressed Fluc, 1 × 10^5^ cells (*n* = 4) were plated in opaque flat bottom 96 well plates (Thermo Fisher Scientific, MA) in triplicate for 24 hours before being imaged. Prior to imaging on the Xenogen IVIS spectrum in vivo imaging system, XenoLight d‐luciferin potassium salt (PerkinElmer, MA) in CGM was added at 150 μg/mL. A photographic image of the plate followed by a luminescent image was recorded. For quantification, the intensity of the luminescent signal in each well was recorded as total flux (average photons per second, p/s).^50^ Images were analyzed using the Living Image 4.3.1 software (PerkinElmer).

### 2.3 Immunophenotypic assessment by flow cytometry

Immunophenotyping was done on batches of isolated ASCs (*n* = 6) before and after transduction. The following monoclonal antibodies were used: Armenian hamster anti‐mouse/rat CD29 APC and IgG isotype control APC (1.25 μL), mouse anti‐rat CD45 APC‐eFluor780 and IgG1 K isotype control APC‐eFluor780 (2 μL), mouse anti‐mouse/rat CD90.1 PE‐Cyanine 7 and IgG2a K isotype control PE‐Cyanine 7 (1 μL; eBioscience, ThermoFisher Scientific, MA), and mouse anti‐rat CD31 PE and IgG1, κ isotype control PE (3 μL; BD Biosciences, CA). A 100 μL cell aliquot containing at least 1 × 10^5^ viable cells was incubated in the dark (15 minutes, 37°C) after adding the four monoclonal antibodies (CD29, CD45, CD90, and CD31). Following incubation, cells were washed thrice with phosphate‐buffered saline (PBS, Gibco, Life Technologies) supplemented with 2% FBS, resuspended in PBS and then analyzed for antigen expression. A single tube containing unstained cells and a tube stained with the isotype controls were prepared for every sample to verify protocol settings and to serve as a negative control. Data were acquired on a Gallios flow cytometer (Beckman Coulter, California). To determine the percentage transduced cells, GFP expression was measured together with the surface markers. The viability stain 4′,6‐diamidino‐2‐phenylindole (DAPI, Beckman Coulter) was included to allow analysis only of living cells. Data analysis was performed using Kaluza Flow Cytometry analysis software 1.3 (Beckman Coulter).

### 2.4 In vitro differentiation capacity of ASCs

To confirm the differentiation capacity of non‐transduced and transduced ASCs, they were induced to differentiate along adipogenic and osteogenic lineages using specific induction media. Cell cultures were trypsinized, counted, phenotypically characterized, and replated into 24‐well microplates (iBidi, Martinsried, Germany) at 5 × 10^3^ cells/cm^2^. Adipogenic induction medium consisted of CGM supplemented with 0.1 μM dexamethasone, 0.5 mM 3‐isobutyl‐methylxanthine (IBMX), 56 μM indomethacin, and 10 μM insulin (human recombinant zinc, Gibco, Life Technologies). Osteogenic induction medium consisted of CGM supplemented with 10 nM dexamethasone, 50 μM ascorbate‐2‐phosphate, and 10 mM β‐glycerophosphate. Stock solutions of dexamethasone and indomethacin were prepared in ethanol, whereas IBMX stock solutions were prepared in dimethyl sulfoxide (DMSO). Non‐induced control cells were maintained in CGM. After 14 or 21 days in either induction medium or CGM, cultures were fixed (60 minutes) using 4% (vol/vol) formaldehyde solution. The accumulation of lipid droplets was detected by staining the cultures with a 0.3% oil red O (ORO, 10 minutes, RT) after 14 days of induction. Mineralization was detected by staining the cultures with 2% alizarin red S (ARS, pH 4.1, 30 minutes, RT) 21 days after induction. Both the differentiated and control cells were stained. Images were acquired using an Eclipse Ts2‐FL inverted microscope fitted with a DS‐Fi3 camera and DS‐L4 control unit (Nikon corporation, Tokyo, Japan). Dexamethasone, IBMX, indomethacin, ascorbate‐2‐phosphate, β‐glycerophosphate, DMSO, formaldehyde solution, ORO, and ARS were all from Sigma–Aldrich Chemie, Steinheim, Germany.

### 2.5 In vivo rat model and ASC administration

**Figure 1:**
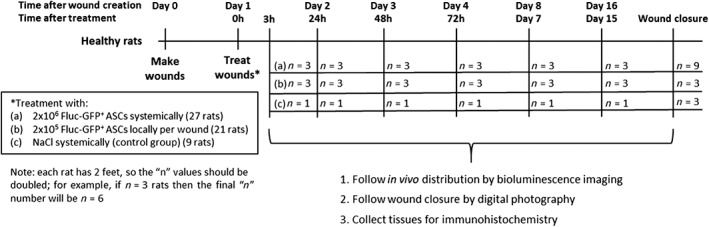
Study design. For the rat model of wound repair under physiological conditions, full‐thickness wounds were created bilaterally on the dorsal aspect of the hind paws. One day after wounding, animals were treated either with a single injection of 2 × 10^6^ ASCs systemically into the tail vein or locally with 2 × 10^5^ ASCs in total, split into two sides of each wound. Control animals received a single injection of NaCl systemically into the tail vein. Animals were followed by digital photography and bioluminescence imaging and sacrificed for the collection of tissues for histology at 3 hours, 24 hours, 48 hours, 72 hours, 7 days, and 15 days post‐treatment and at wound closure. Abbreviations: ASCs, adipose‐derived mesenchymal stromal cells; Fluc, firefly luciferase; GFP, green fluorescent protein

Female Wistar rats (≥10 weeks old, ≥250 g) were fed a standard diet and given water ad libitum. For all procedures, animals were anesthetized by inhalation of 3%–5% isoflurane (Baxter AG, Opfikon, Switzerland). Wounds on the dorsal aspect of the hind paws were created bilaterally by removing a full‐thickness skin area of 1.2 cm × 0.8 cm. One day after wounding, 27 animals received a single injection of 2 × 10^6^ Fluc‐GFP positive ASCs systemically into the tail vein and 21 animals locally with 2 × 10^5^ Fluc‐GFP positive ASCs in total, split between two sides of each wound (proximally at the ankle and distally at the toes). One day post wound creation was chosen for administration so that the inflammatory phase would already be initiated to attract the ASCs. Moreover, by postponing the injection of the ASCs, we avoided the probability of the cells being washed away due to bleeding immediately after wound creation. For each experiment, ASCs isolated from two rats were cultured and transduced separately before being pooled for either systemic or local administration. Nine control animals received a single injection of sodium chloride (NaCl) systemically into the tail vein. Both feet were evaluated separately for each animal. All animals received a semi‐occlusive wound dressing that was changed at every evaluated time‐point (Figure 1).

### 2.6 In vivo tracking of administered ASCs by BLI

Animals treated systemically and locally were imaged by BLI at 3 hours, 24 hours, 48 hours, 72 hours, d7, d15, and at WC. Prior to imaging, animals received a single IP injection of firefly d‐luciferin potassium salt (150 mg/kg body weight) prepared in PBS. After 20 minutes, imaging was performed using the Xenogen IVIS spectrum in vivo imaging system with an XGI‐8 Gas anesthesia system. A photographic image of the animal followed by a luminescent image was recorded by the camera. To exclude background luminescence emitted from the ASCs themselves, a single animal that received non‐transduced ASCs at the same concentration and mode of administration was included as a control for every BLI experiment. For quantification, three regions of interest (ROI) were manually selected to quantify the luminescent signal in the lung (ROI 1) and the wound area (right foot, ROI 2; left foot ROI 3). The ROI size was kept constant between animals and the intensity of the luminescent signal was recorded as total flux (average photons per second, p/s).^50^ All images were analyzed using the Living Image 4.3.1 software.

### 2.7 Histological assessment of GFP‐positive ASCs

**Figure 2:**
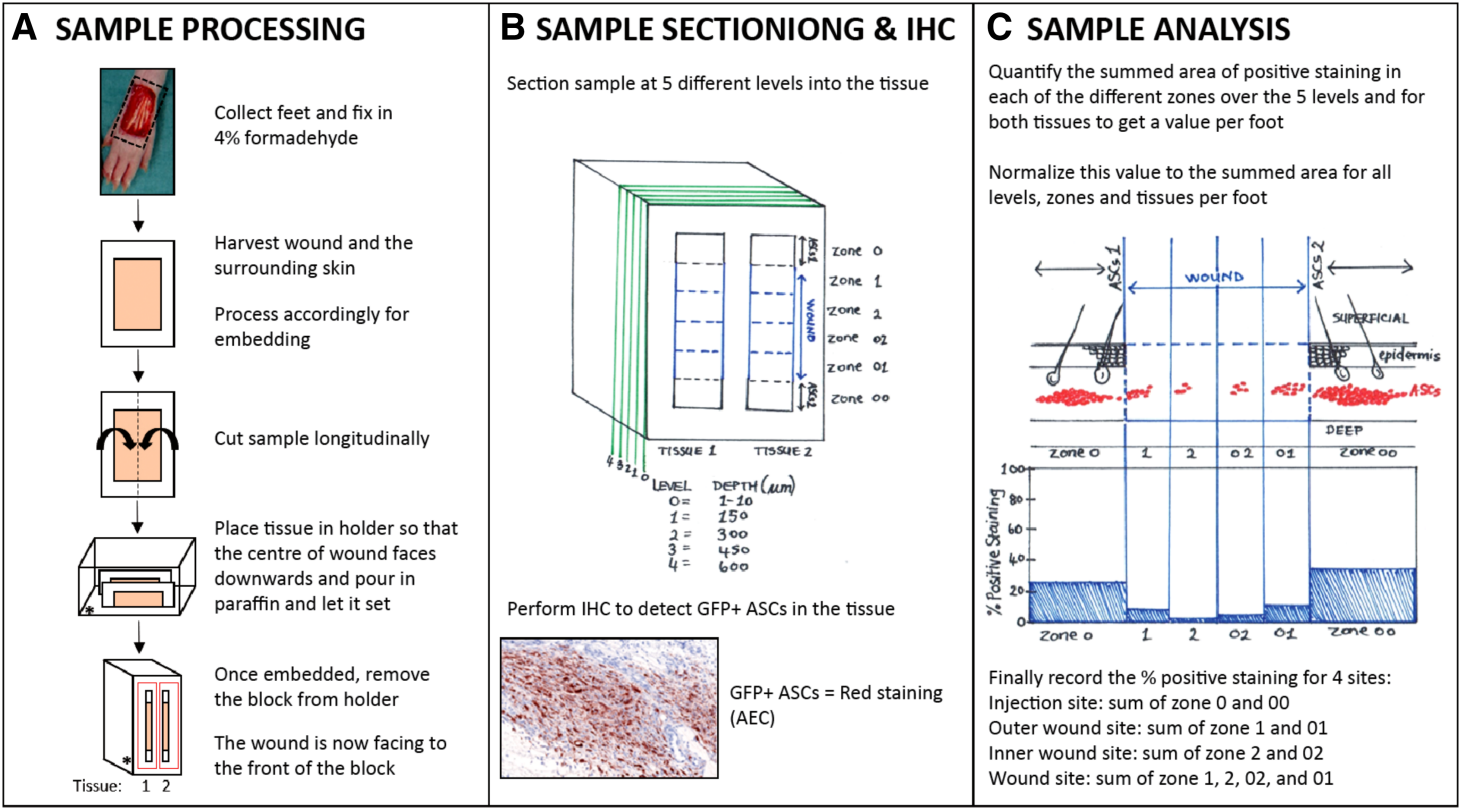
Schematic showing how the locally treated feet samples were processed, sectioned, stained for IHC, and analyzed. A, The feet were collected, fixed, and then processed for embedding in paraffin blocks. B, Each block, consisting of a single sample, was sectioned at five different levels into the FFPE tissue at levels 0, 1, 2, 3, and 4. IHC was performed to detect GFP^+^ ASCs (red staining, AEC). C, The sections were analyzed to quantify the area of positive staining using image processing software. Each section was divided into six zones, where zone 0 = ASCs 1 (ASC injection site 1), zone 1, 2, 02, and 01 = wound divided into four equal regions, and zone 00 = ASCs 2 (ASC injection site 2). The summed area of positive staining in each of the different zones over the five levels and for both tissues was quantified to obtain a value per foot. This value was normalized to the summed area for all the levels, zones, and tissues per foot. Finally, the percentage of positive staining was recorded as four sites: the injection site (sum of zone 0 and 00), the outer wound site (sum of zone 1 and 01), the inner wound site (sum of zone 2 and 02), and the wound site (sum of zone 1, 2, 02, and 01). Abbreviations: IHC, immunohistochemistry; FFPE, formaldehyde fixed paraffin embedded; ASCs, adipose‐derived mesenchymal stromal cells; AEC, aminoethyl carbazole; GFP, green fluorescent protein

For systemically and locally treated groups, the lungs and the entire foot with the wound and the surrounding uninjured skin were harvested at 3 hours, 24 hours, 72 hours, day 7 (d7), d15, and at WC and fixed in 4% formaldehyde (Merck Millipore, Massachusetts). After fixation, the wound area along with the uninjured surrounding skin was removed from the foot and processed for paraffin embedding (Figure 2A). The lungs were kept whole and processed for paraffin embedding.

For immunohistochemistry (IHC), 5 μm thick transverse tissue sections were cut from formaldehyde fixed paraffin embedded (FFPE) samples and analyzed using an anti‐GFP D5.1 XP rabbit monoclonal antibody (Cell Signaling Technology Inc., Massachusetts). A single section of the lungs and feet was analyzed for the systemically treated group. For the locally treated group, a single section of the lungs was analyzed. However, for the feet, tissue sections were analyzed at five different levels into the sample block (Figure 2B). Antigen retrieval was performed using the Pascal Citrate system (DakoCytomation, Hamburg, Germany). After blocking endogenous peroxidases with Dako REAL peroxidase blocking solution (Dako, Hamburg, Germany), sections were incubated with the primary GFP rabbit monoclonal antibody (1/100 dilution; 70 ng/mL) for 1 hour at RT, and then with a horseradish peroxidase‐(HRP) complex secondary antibody (Dako EnVision+System‐HRP, Dako, Hamburg, Germany) for 30 minutes at RT. Sections were developed with aminoethyl carbazole (AEC) for 10 minutes (BioGenex, California) and counterstained with hematoxylin for 1–2 minutes. Sections were scanned using a Zeiss Axio Scan Z1 Brightfield scanner (Carl Zeiss, AG, Germany) at ×20 magnification.

In the locally treated group, we determined the precise location of the administered GFP positive ASCs over time. To do so, each section from the five different levels for each FFPE sample was divided into six zones: zone 0 = ASCs injection site on the border of wound; zone 00 = ASCs injection site on the other border of wound; and the wound itself divided in four zones: zone 1, 2, 02, and 01 (Figure 2C). The location of ASCs in the wound was expressed as a percentage area of GFP staining for a given zone and foot for each time‐point. Using these zones, the percentage area of GFP staining was recorded at 24 hours, 48 hours, 72 hours, and d7 as four sites, summing up all the five levels together as a single value per foot. The injection site was determined as the sum of zone 0 and 00 and the wound site as the sum of zone 1, 2, 02, and 01. More precisely, the outer wound site was defined as the sum of zone 1 and 01, and the inner wound site as the sum of zone 2 and 02 (Figure 2C). In total, three animals each with two feet per animal per timepoint were analyzed (*n* = 6). All images were processed using Definiens 2.7 software (Munich, Germany). Results were compiled in the MATLAB 2018b (MathWorks, MA) and the graphs plotted using either MATLAB or Prism (GraphPad, CA).

### 2.8 Assessment of wound repair

The time taken to complete WC and the contraction/re‐epithelialization ratio were assessed as previously described.^51^ Wound size was documented immediately after wounding and until WC by digital photography at a constant distance with a ruler next to the wound for scaling. The photos were analyzed using ImageJ 1.48v software^52^ to determine the wound area. At complete WC (ie, full epithelialization), the surface of the scar with hairless skin was measured and considered to correspond to the area of the wound that had healed by re‐epithelialization. The surface of the wound that healed by contraction was estimated by subtraction of the epithelialized surface from the original wound area measured at day 0 (Figure 7C).

### 2.9 Statistical analysis

Data are expressed as means ± SD. Statistical analyses were carried out using Prism (GraphPad, California). A Mann‐Whitney test was used to assess differences between data means. Grouped data were assessed by performing multiple *t* tests using the Holm‐Sidak method. A *P* value of <−.05 between data means was considered significant.

## 3 RESULTS

### 3.1 Transduced ASCs maintained their immunophenotype and differentiation capacity

**Figure 3:**
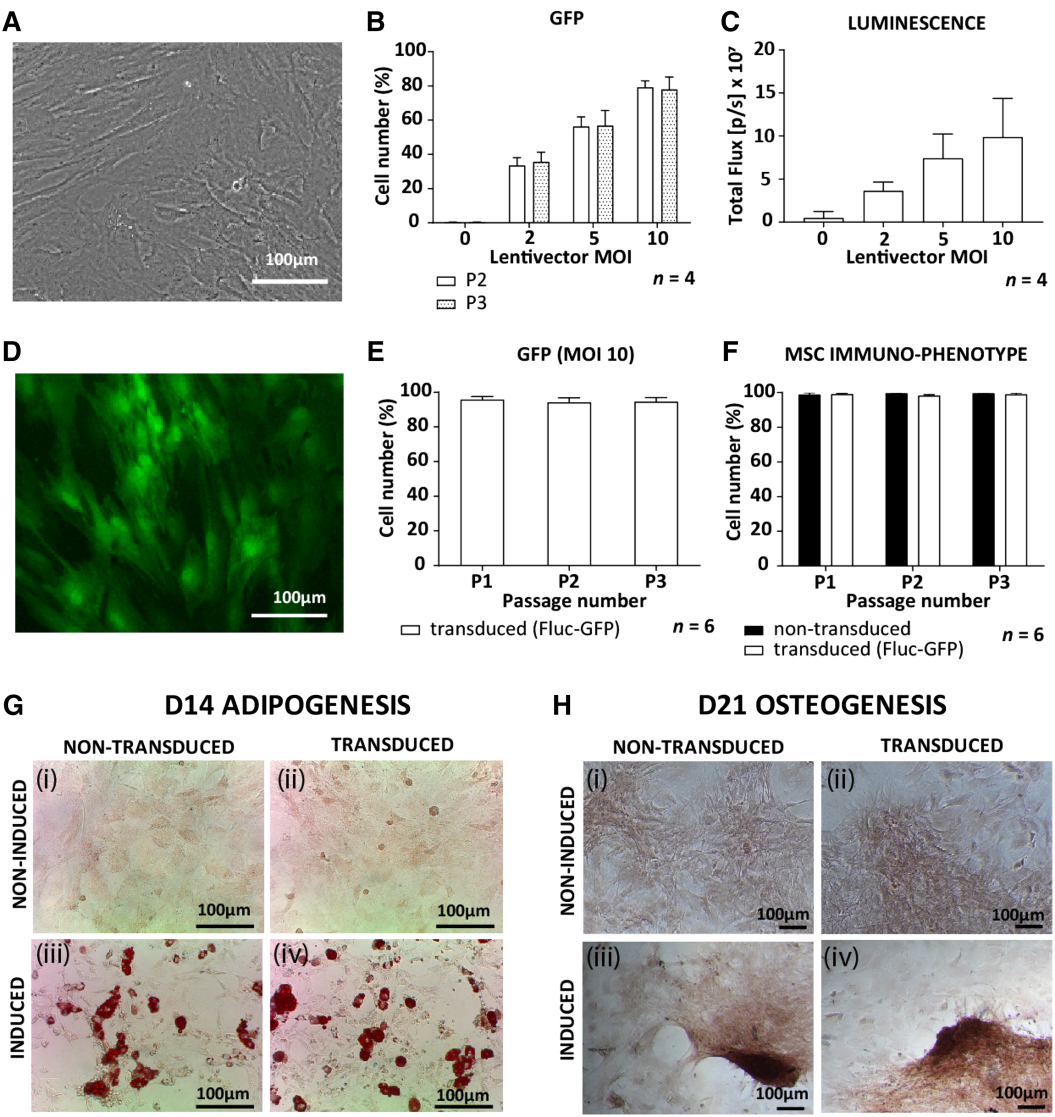
Characterization of non‐transduced and transduced rat ASCs. A, Brightfield image of non‐transduced ASCs in culture. B, GFP expression of transduced ASCs measured by flow cytometry with increasing MOIs as well as, C, luminescence measured by *in vitro* bioluminescence imaging. D, A fluorescent image of transduced ASCs (MOI 10) expressing GFP. E, GFP expression by ASCs transduced with an MOI of 10 in culture from passage 1 to 3 (P1 to P3). F, MSC immunophenotype (CD90+, CD29+, CD45−, CD31−) for transduced and non‐transduced ASCs. G, Qualitative assessment of adipogenesis for (i, ii) non‐induced control ASCs, (iii, iv) induced ASCs, (i, iii) non‐transduced ASCs, and (ii, iv) transduced ASCs all stained for oil red O to identify lipid droplet formation after 14 days in induction medium. H, Qualitative assessment of osteogenesis for (i, ii) non‐induced control ASCs, (iii, iv) induced ASCs, (i, iii) non‐transduced ASCs, and (ii, iv) transduced ASCs all stained for alizarin red S to confirm calcium deposition after 21 days in induction medium. Abbreviations: ASCs, adipose‐derived mesenchymal stromal cells; GFP, green fluorescent protein; MOI, mode of infection

Isolated ASCs were plastic adherent in culture and displayed a typical fibroblast morphology (Figure 3A). Evaluation of GFP expression established that the lowest MOI needed to transduce at least 70% of the ASCs was 10 (Figure 3B). As the Fluc gene is under the control of a different promoter than the GFP gene, we determined whether our newly transduced cells also expressed Fluc. When d‐luciferin, the molecular substrate for firefly luciferase, was added to the transduced cells *in vitro*, emitted luminescence was detected. An increase in luminescence, quantified as total flux, was observed with increasing MOIs (Figure 3C), confirming that the cells were successfully transduced to express not only GFP but also Fluc. GFP fluorescence was visible in both the nucleus and the cytoplasm of the transduced cells (Figure 3D). The percentage cells expressing GFP was also maintained *in vitro* over different passages showing that greater than 94% of ASCs expressed GFP (Figure 3E). Over the three passages evaluated, both transduced and non‐transduced ASCs maintained a typical MSC immunophenotype, being strongly positive for CD90 and CD29 and negative for CD45 and CD31 (Figure 3F). When treated with control medium, the non‐transduced and transduced ASCs showed no spontaneous differentiation as seen by the absence of intracellular lipid droplets or calcium deposition. With induction media, the non‐transduced and transduced ASCs showed comparable levels of intracellular lipid droplet formation, confirming that adipogenesis had occurred. In addition, sparsely distributed cell clumps or nodules that stained positive for ARS were observed, confirming that osteogenesis had occurred (Figure 3G,H).

### 3.2 Systemically administered ASCs were filtered out in the lungs without homing to the wound site

**Figure 4:**
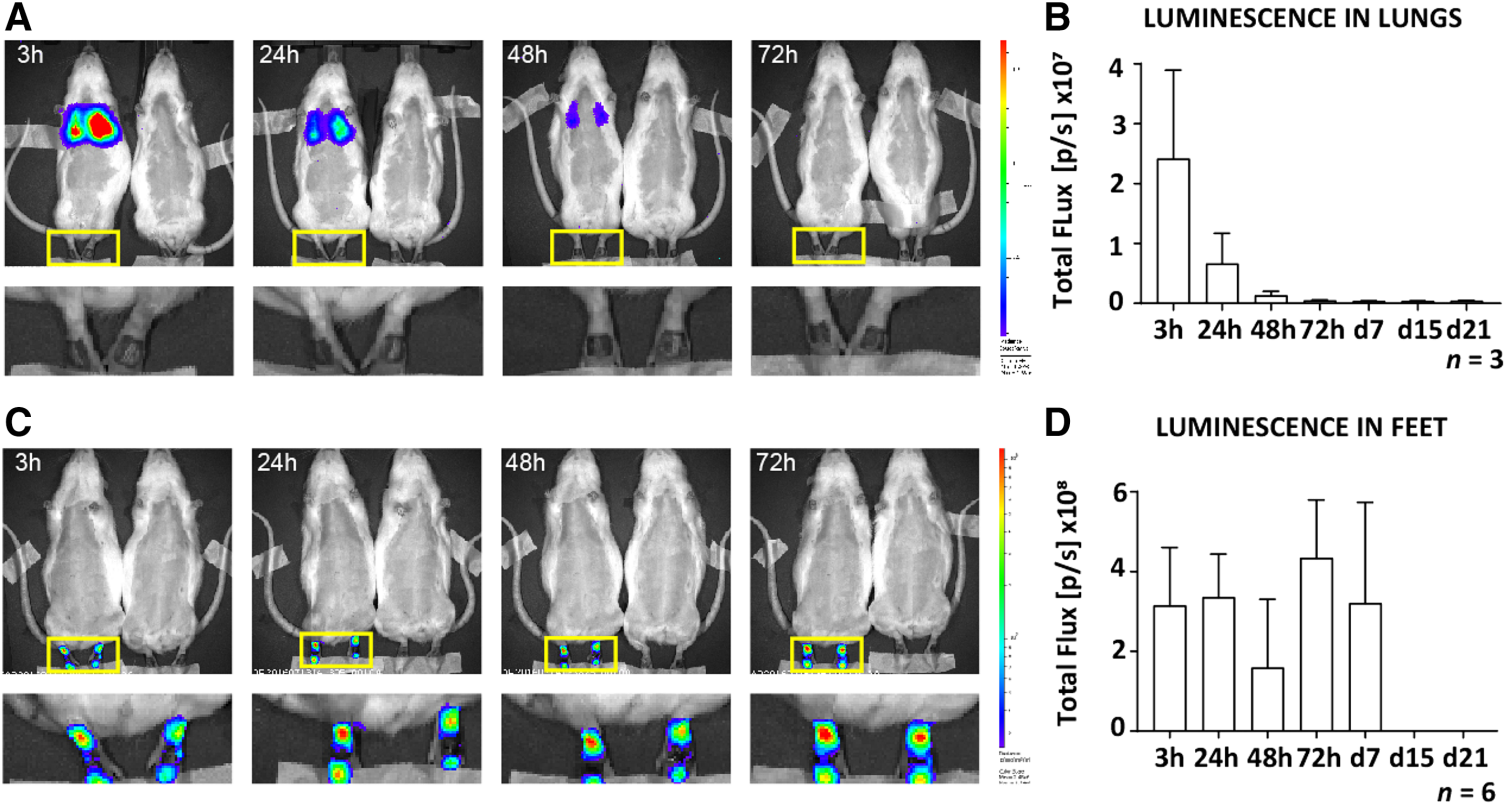
In vivo tracking of systemically and locally administered Fluc‐GFP^+^ ASCs during wound repair under physiological conditions. ASCs transduced to express both Fluc and GFP were injected either systemically (2 × 10^6^ ASCs) via the tail vein or locally (2 × 10^5^ ASCs) into two sides around each wound in rats 24 hours after bilateral full‐thickness wounds had been created on the dorsal aspect of the feet. Prior to imaging, rats received 150 mg/kg body weight of d‐luciferin. A single representative image of a, A, systemically and, C, locally treated rat and BLI control rat (received non‐transduced cells) imaged at 3, 24, 48, and 72 hours. An enlarged area of the feet of the treated animals is also shown. The luminescence signal in the, B, lungs of systemically treated and the, D, feet of locally treated rats was quantified and recorded as total flux over time (gray bars). Data shown as mean ± SD. Abbreviations: ASCs, adipose‐derived mesenchymal stromal cells; BLI, bioluminescence imaging; Fluc, firefly luciferase; GFP, green fluorescent protein

**Figure 5:**
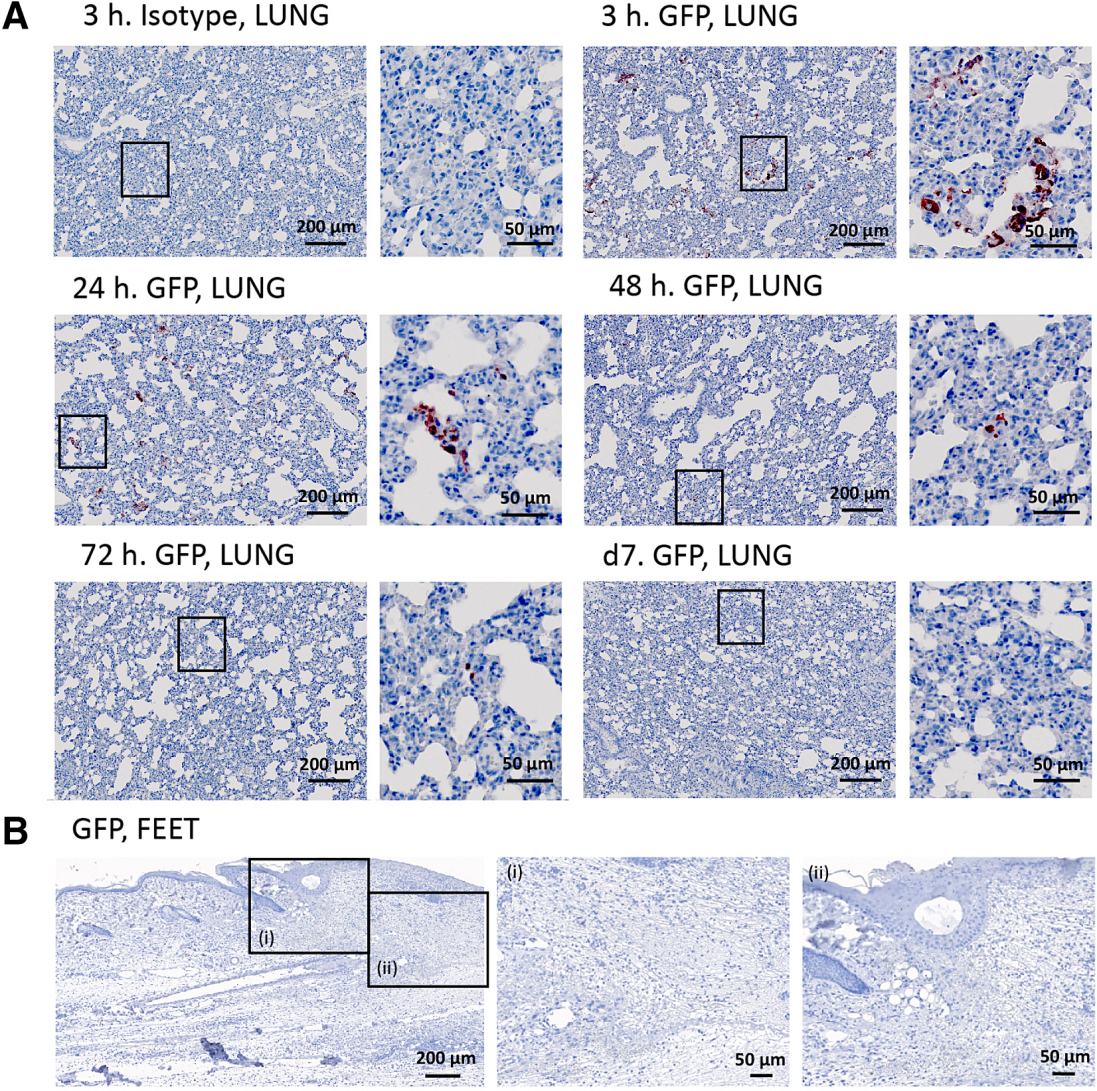
Detection of systemically administered ASCs during physiological wound repair by IHC. ASCs transduced to express both Fluc and GFP were injected systemically (2 × 10^6^ ASCs) via the tail vein in rats 24 hours after bilateral full‐thickness wounds had been created on the dorsal part of the feet. Lungs and feet were processed for IHC to detect GFP positive ASCs (red staining, AEC). A, Images of the lung at 3 hours, 24 hours, 48 hours, 72 hours, and at d7 are shown. The right panel is a magnified view of the black rectangle showing positively stained ASCs. B, A representative image of the feet is shown where panel (i) is a magnified view of the epithelium lip and (ii) of the wound bed. Tissue sections were counterstained with hematoxylin (blue staining). Abbreviations: AEC, aminoethyl carbazole; ASCs, adipose‐derived mesenchymal stromal cells; Fluc, firefly luciferase; GFP, green fluorescent protein; IHC, immunohistochemistry

Following systemic administration, the luminescent signal was detectable in the lungs 3 hours post administration (Figure 4A), and a decrease in signal was observed from 3 to 72 hours (Figure 4B). No signal was detected in the wound on the feet. No signal was detectable in other organs when ASCs were injected either systemically or locally. At the histological level, systemically administered GFP positive ASCs were easily detected in the lungs of injected animals at 3 and 24 hours, moderately detectable at 48 hours, barely detectable at 72 hours, and undetectable from day 7 (Figure 5A). No GFP staining was detected in the wounds on the feet (Figure 5B) or in other non‐target organs such as the spleen, heart, kidney, and liver at any of the time‐points (data not shown).

### 3.3 Locally administered ASCs survived and became distributed within the wound bed

**Figure 6:**
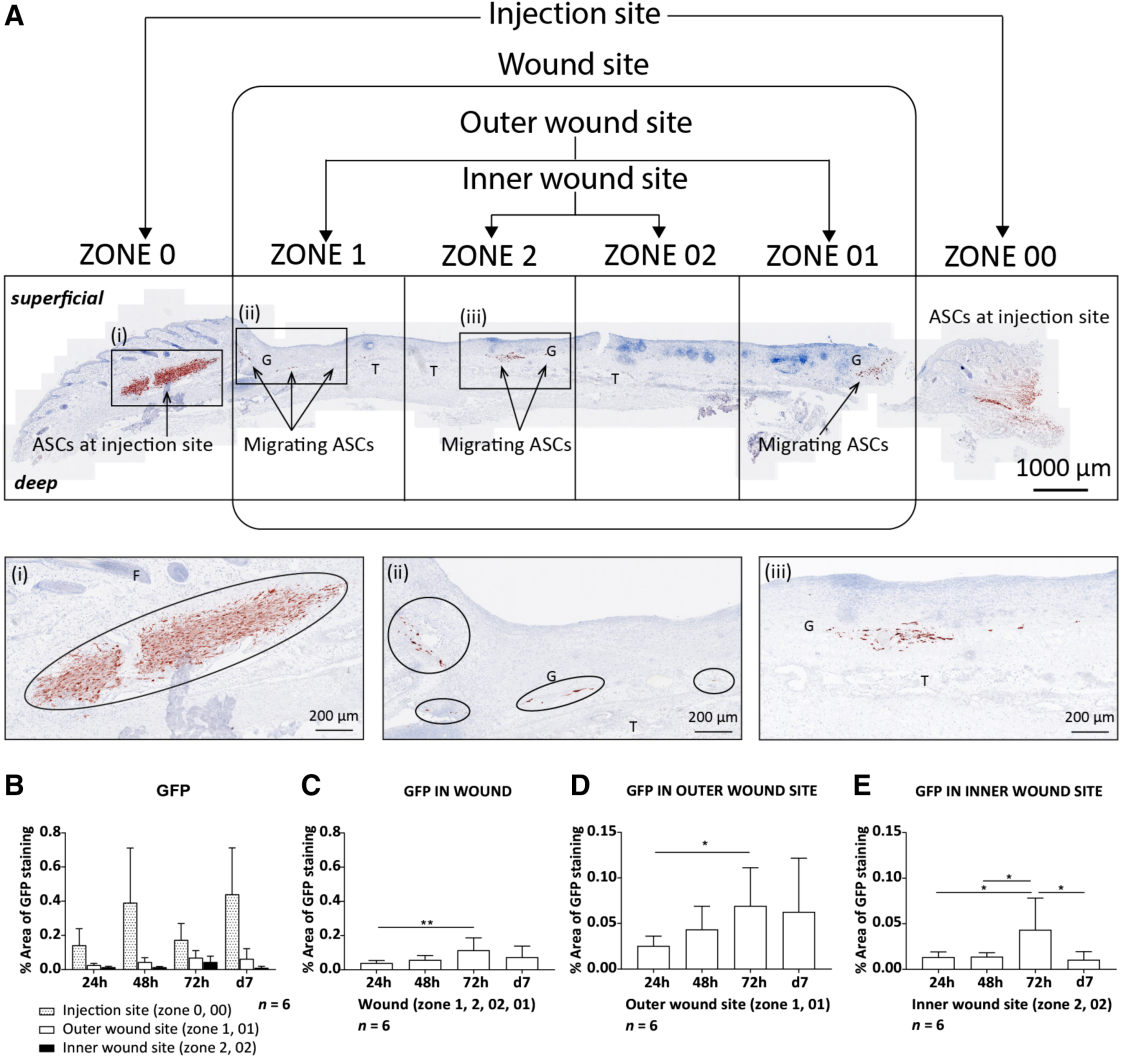
Locally administered ASCs were detected within the wound over time. A total of 2 × 10^5^ ASCs transduced to express both Fluc and GFP were injected locally into two sides around each wound 24 hours after bilateral full‐thickness wounds had been created on the dorsal part of the feet. A, a single tissue section from the wound in the feet 24 hours post injection stained for GFP (red staining, AEC). Magnified images of the (i) injection site, (ii) outer wound site, and (iii) inner wound site. The distribution of ASCs at the different sites of the wound was quantified over time showing, B, The injection site, outer, and inner wound site in a single graph, C, the wound site (zone 1, 2, 02, 01), D, the outer wound site (zone 1, 01), and, E, the inner wound site (zone 2, 02). Data shown as mean ± SD. Significance is shown where *P < .05; **P < .01. Abbreviations: AEC, aminoethyl carbazole; ASCs, adipose‐derived mesenchymal stromal cells; E, epithelium; F, hair follicles; Fluc, firefly luciferase; G, granulation tissue; GFP, green fluorescent protein; T, tendons

When administered locally, the luminescent signal remained strongly detectable for 7 days at the injection site (Figure 4C,D) with no signal being detectable in the lungs (Figure 4C). Interestingly, histological analysis of animals that received ASCs locally revealed that GFP staining was not restricted to the ASC injection sites. When 15% of the wound was analyzed as represented by the five levels at which sections were assessed, GFP positive ASCs became distributed within the wound bed as early as 24 hours after administration (Figure 6A‐E). At the injection site (zone 0, 00), ASCs remained detectable until d7 (Figure 6B). Within the wound bed (zone 1, 2, 02, and 01), a significant increase in GFP staining was found from 24 to 72 hours (0.039% vs 0.057%, *P* = .0087) with no significant change on d7 (Figure 6C). This significant increase in GFP staining from 24 to 72 hours was found in both the outer (zone 1 and 01; 0.031% vs 0.047%, *P* = .0043) and inner wound sites (zones 2 and 02; 0.010% vs 0.033%, *P* = .0173). However, even though the increase was significant in the inner wound site between 24 and 72 hours (0.013% vs 0.043%, *P* = .0173), and 48 and 72 hours (0.014% vs 0.043%, *P* = .0152), GFP staining remained lower than in the outer wound site. The outer wound site showed no significant changes in GFP staining from 72 hours to d7, whereas in the inner wound site a significant decrease was found from 72 hours to d7 (0.043% vs 0.010%, *P* = .0152; Figure 6D,E).

### 3.4 Both systemic and local administration lead to enhanced WC time

**Figure 7:**
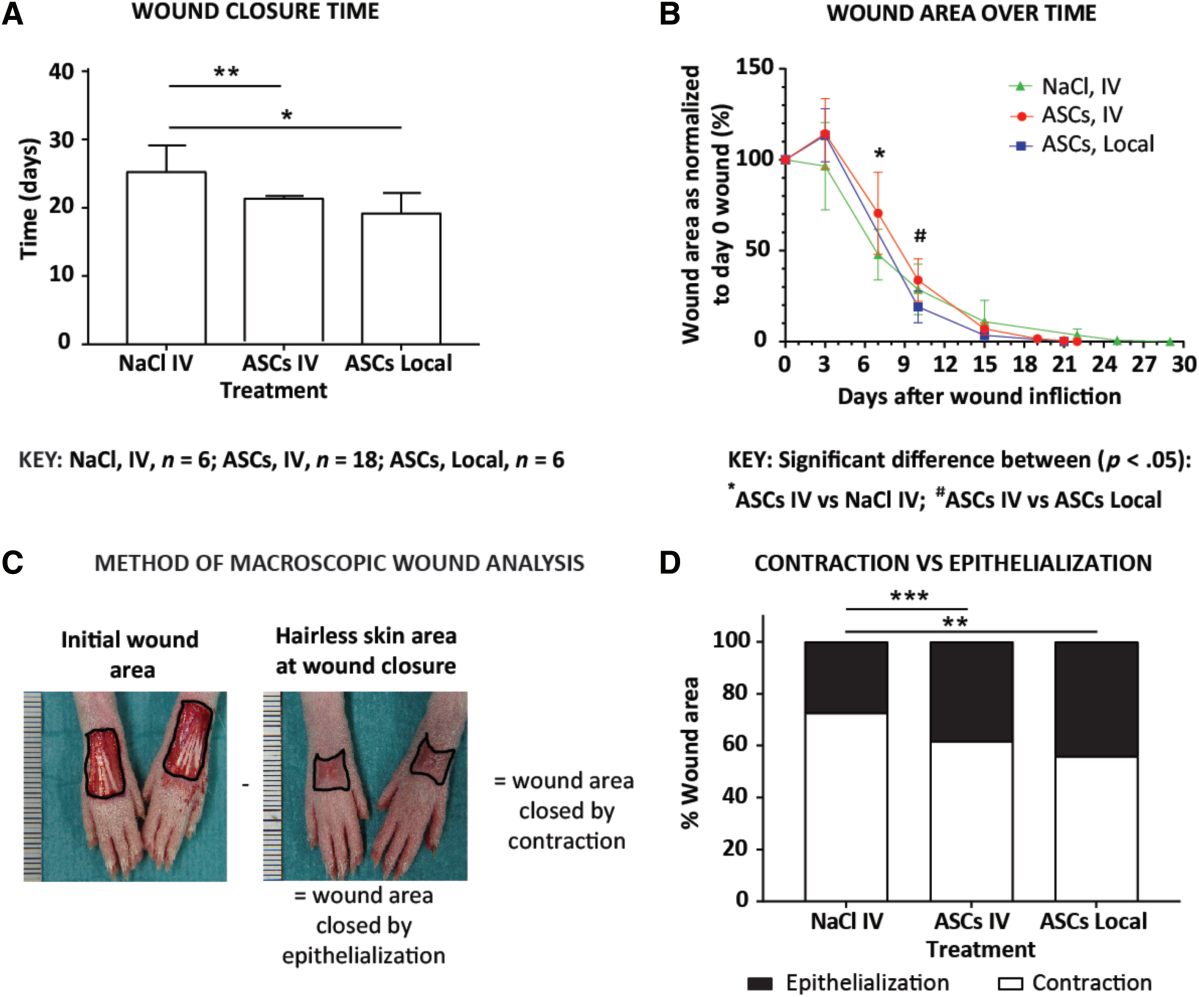
Wound closure assessment in animals treated locally and systemically. Animals were treated with Fluc‐GFP^+^ ASCs, injected locally into two sides around each wound (2 × 10^5^ ASCs) or systemically (2 × 10^6^ ASCs) into the tail vein. Control animals were treated systemically with NaCl. A, Time taken for complete wound closure after treatment. B, Wound area over time after treatment normalized to the original wound area on day 0. C, Calculation of the contraction/epithelialization ratio: the area of the wound is measured directly after surgery. At the day of complete wound closure, the area of hairless skin is measured and considered to correspond to the area of the wound closed by epithelialization. Subtracting the area of epithelialization from the initial wound area provides the area closed by contraction. D, Wound area closed by contraction and by epithelialization. Data shown as mean ± SD. Significance is shown where *P < .05; **P < .01; ***P < .001. Abbreviations: ASCs, adipose‐derived mesenchymal stromal cells; Fluc, firefly luciferase; GFP, green fluorescent protein

As a secondary aim, we determined whether ASC administration might lead to a change in WC time. In comparison to a total WC time of 26 days in the NaCl treated group, WC was significantly faster when ASCs were administered locally (19 days, *P* = .0108) and systemically (21 days, *P* < .0024; Figure 7A). Both ASC treated groups showed an increase in wound size after 3 days before reducing in size. At 7 days, wound size was significantly smaller in the control group compared with the ASC systemically treated group (*P* = .03013); however, this was not maintained. Comparison of ASC treated groups showed significantly smaller wounds with local treatment at 10 days (*P* = .0102; Figure 7B). Further investigation into whether WC was favored more by contraction (C) or epithelialization (E) showed a significant decrease in C and an increase in E in both the locally (C: 56%, E: 44%) and systemically treated groups (C: 62%, E: 38%) compared with the NaCl treated group (C: 73%, E: 27%; *P* = .0022 and *P* = .0002, respectively; Figure 7C,D).

## 4 DISCUSSION

Interest in the use of systemically applied MSCs for therapeutic purposes was popularized by the group of Horwitz. Their studies in children with osteogenesis imperfecta demonstrated that allogeneic BM‐MSCs injected systemically engrafted in the bone and BM stroma, and led to improvement in growth rates of the children as well as their ability to synthesize intact bone.^53^, ^54^, ^55^ A study in rats showed that BM‐MSCs administered either systemically or locally were able to promote fracture healing.^56^ Although the proportion of BM‐MSCs that migrated into the injury sites following systemic injection was significantly less compared with locally injected BM‐MSCs, both groups promoted fracture healing equally well, indicating that systemically injected BM‐MSCs may have contributed in an indirect manner.^56^

We set out to compare the fate and distribution of systemically and locally administered ASCs in an in vivo model of physiological wound repair. The first concern with using transduced ASCs was that this could change their characteristics. Two parameters currently used to identify MSCs were assessed, namely their immunophenotype and differentiation capacity.^30^, ^57^ Transduction of ASCs did not change their immunophenotype or differentiation capacity compared with non‐transduced ASCs and transduced ASCs maintained GFP expression *in vitro* over several passages. This confirmed previous studies showing that MSCs transduced to express GFP^58^, ^59^ or Fluc^33^ maintain their characteristics *in vitro*. Here, we report that by using a single lentivector, both GFP and Fluc can be used in combination without changing the characteristics of ASCs.

Systemically administered ASCs showed no detectable homing to the site of injury. We suggest two reasons for this: first, ASCs could not bypass entrapment in the lung and re‐enter the circulation; and second, the inflammatory response from the site of injury was not strong enough to activate ASC homing during physiological wound repair. We postulate that pulmonary passage was the major reason for the lack of detectable ASC homing. Entrapment in the lungs has been a recurring problem with systemic administration of MSCs.^60^, ^61^ Of note, lung entrapment in our rat model was not associated with any visible signs of distress or deterioration of health.

To overcome this pulmonary first‐pass effect and to ensure that administered cells reach the wound bed at the injured site in a timely manner and in sufficient numbers, alternative routes of administration have been suggested such as regional or local injection.^27^, ^29^, ^55^ We investigated the fate of locally administered ASCs and found that they remained viable at the injection sites for 7 days. This is in line with another study reporting that rat BM‐MSCs transplanted into traumatized skeletal muscle could be tracked in vivo until day 7.^33^ At the histological level, we found a significant increase in ASCs localized within the wound as opposed to the adjacent injection sites from 24 to 72 hours. This localization of ASCs could be explained by active ASC migration, ASC proliferation, or the passive displacement from the injection site into the wound bed as the wound contracts to bring the wound edges closer together. Over time, more ASCs were found in the outer wound site close to the newly extending epithelium lip at all time‐points evaluated, with the greatest number of ASCs being detected at 72 hours. In the inner wound site, increased numbers of ASCs were detected at 72 hours. Our data suggest that locally administered ASC have the capability to migrate within the wound bed.

Wounds can close by two mechanisms, contraction, or re‐epithelialization. Contraction occurs predominately as a rapid repair mechanism, whereas re‐epithelialization takes longer and is closer to regeneration. Our animal model allowed for measurements of wound size, time to complete healing, and ratios of WC by contraction and epithelialization. Interestingly, an enhancement in WC time was found with ASC treatment independently of the route of administration. Both administration routes resulted in more rapid WC by re‐epithelialization rather than contraction as compared with the control group. The enhancement of WC by re‐epithelialization rather than contraction is of clinical relevance, as it can have a significant impact on the quality of the scar by decreasing scar retraction.

The disappearance of transplanted cells and low engraftment at the injured site while still enhancing WC time suggests that ASCs play a role as initiators of wound repair rather than effectors.^62^ This confirms findings from a study using a mouse model of acute acid‐burn injury.^63^ The authors also found that despite short‐lived survival of locally transplanted ASCs, WC was enhanced. Another study found that intravenous human MSCs improved myocardial infarction in mice without significant engraftment through the secretion of anti‐inflammatory protein TSG‐6.^64^ The mechanism of wound repair by MSCs has been suggested to stem from their paracrine signaling,^65^ which mobilizes the host cells to promote wound repair. This leads us to ask; how do MSCs filtered out in the lung influence wound repair mechanisms that are located distally? It has been shown that MSCs secrete various molecules that are modulators of cellular growth, replication, differentiation, and adherence.^66^, ^67^ The MSC (BM‐MSCs and ASCs) secretome consists of soluble factors, such as cytokines, chemokines and growth factors, and proteins, lipids, and nucleic acids that are released via extracellular vesicles.^68^, ^69^ A subset of these extracellular vesicles, referred to as exosomes, are believed to be released by MSCs trapped in the lungs in response to injury cues where they can travel via the circulation to the injured site to exert their therapeutic effects.^55^, ^70^ MSC‐derived extracellular vesicles have been suggested to be partially responsible for this paracrine effect.^71^ It has been shown that systemically administered MSC exosomes attenuated lesion size and improved functional recovery post spinal cord injury.^72^ A study that examined the role of MSC exosomes in wound healing showed that these structures were able to induce proliferation and migration of fibroblasts as well as enhance angiogenesis *in vitro*.^73^

This study confirms that systemically injected ASCs behave in a manner similar to BM‐MSCs, being able to enhance WC despite showing intrapulmonary trapping and limited homing to the wound area. We provide evidence of migration of locally injected ASCs into the wound bed. We suggest that the therapeutic effect of ASCs is due to their paracrine signaling. Clinical studies using ASCs for cutaneous non‐healing/chronic wounds are still limited. Instead, animal models are used. The local administration of ASCs for wound repair is favored and is being evaluated for various injuries with/without scaffolds in animal studies of tendon repair,^74^, ^75^, ^76^ cutaneous non‐healing wounds,^77^, ^78^, ^79^, ^80^, ^81^, ^82^, ^83^ burn injuries,^84^ spinal cord injuries,^85^, ^86^ and for bone defects,^87^ among other indications. In the field of plastic and reconstructive surgery, since the discovery of ASCs within adipose tissue,^4^, ^88^ the addition of both the SVF and ASCs have been investigated in order to overcome the re‐absorption of fat grafts.^89^, ^90^, ^91^

A limitation of our work could be the underestimation of ASC homing and engraftment because of our detection techniques and experimental setup. Although BLI allows for in vivo tracking, single cells and luminescence in deep organs cannot be detected. To overcome this, we included GFP staining to detect ASCs at a single cell level and in non‐target organs. The animal model may have influenced the experimental outcome since Wistar rats are outbred and thus ASCs were isogenic rather than autologous. The “foreign” or isogenic ASCs might have primed the immune system and enabled a faster response and healing effect. In future studies, the use of alterative systemic routes such as intra‐arterial to overcome the pulmonary first‐pass effect, or treatment to prevent such filtering for example, by vasodilation, should be considered.^55^ Another avenue to explore is the repeated administration of systemic ASCs. Fischer and colleagues hypothesized that the increase in pulmonary passage seen with their study was due to saturation of receptors during the first treatment, allowing more cells to pass the vasculature with the second treatment.^61^ For local administration, the use of scaffolds or dressings could enable longer viability and more directed administration of ASCs into the wound bed. The lentiviral transduction of ASCs could alter properties that we were unable to detect. This could be a limitation for acceptance by regulatory authorities as proof of principle for advancing to phase I clinical trials. More in depth characterization of ASCs is needed to confirm that transduction does not affect their characteristics in vivo.

In this study, we provide data showing an accelerating effect of ASCs on cutaneous wound repair under physiological conditions. These data support the use of ASCs in wound management since physiological wound repair is generally considered a naturally effective process with little margin for improvement. In order to determine the clinical usefulness of ASC injection in the management of chronic wounds, we plan to perform further investigations studying their effect in a model of wound repair under pathological conditions.

## 5 CONCLUSION

This study demonstrates that although the survival and homing of systemically administered ASCs was limited, they nonetheless led to enhanced wound repair. Locally administered ASCs survived for 7 days at the transplantation site. They showed the ability to migrate within the wound bed, effectively enhancing wound repair by significantly reducing WC time. Taken together, ASCs have the potential to enhance wound repair without the need for homing to the injured site, possibly by paracrine signaling through the release of exosomes.

## CONFLICT OF INTEREST

M.S.P. declared leadership position with Anion Biosciences, researcher funding from South African Medical Research Council, Wellcome Trust and stock ownership with Transcure Bioservices. The other authors indicated no potential conflicts of interest.

## AUTHOR CONTRIBUTIONS

K.K.: conception and design, collection and/or assembly of data, data analysis and interpretation, manuscript writing, final approval of manuscript; D.A.‐L.: conception and design, data analysis and interpretation, manuscript writing, final approval of manuscript; M.B.: collection and/or assembly of data, data analysis and interpretation, final approval of manuscript; K.‐H.K.: provision of study materials, data interpretation, final approval of manuscript; M.S.P., B.P.‐C.: conception and design, financial support, data interpretation, manuscript writing, final approval of manuscript; A.M.: conception and design, financial support, administrative support, data interpretation, manuscript writing, final approval of manuscript.

## Data Availability

The data that support the findings of this study are available from the corresponding author upon reasonable request.
